# Home‐Based Intervention to Prevent Functional Decline in (Pre)frail Older Adults: The PromeTheus Randomized Controlled Trial

**DOI:** 10.1002/jcsm.70306

**Published:** 2026-05-14

**Authors:** Kilian Rapp, Corinna Nerz, Tim Fleiner, Michael Denkinger, Gisela Büchele, Martin Rehm, Benjamin Mayer, Dietrich Rothenbacher, Christian Grüneberg, Tobias Braun, Ingrid Hendlmeier, Martina Schäufele, Judith Dams, Hans‐Helmut König, Clemens Becker, Jürgen M. Bauer, Christian Werner

**Affiliations:** ^1^ Department of Clinical Gerontology Robert‐Bosch‐Hospital Stuttgart Germany; ^2^ Institute for Geriatric Research Ulm University Medical Center Ulm Germany; ^3^ Agaplesion Bethesda Clinic Geriatric Center Ulm Ulm Germany; ^4^ Institute of Medical Engineering and Mechatronics Ulm University of Applied Sciences Ulm Germany; ^5^ Institute of Epidemiology and Medical Biometry Ulm University Ulm Germany; ^6^ Division of Physiotherapy, Department of Applied Health Sciences Hochschule für Gesundheit Bochum (University of Applied Sciences) Bochum Germany; ^7^ Faculty of Health HSD Hochschule Döpfer Cologne Germany; ^8^ Department of Social Work University of Applied Sciences Mannheim Germany; ^9^ Department of Health Economics and Health Services Research University Medical Center Hamburg‐Eppendorf Hamburg Germany; ^10^ Geriatric Center, Medical Faculty Heidelberg Heidelberg University Heidelberg Germany

**Keywords:** activities of daily living, ageing, community health services, exercise, frailty, mobility limitations

## Abstract

**Background:**

Older adults with (pre)frailty are vulnerable to deteriorations in physical functioning, mobility and independence. Evidence for frailty interventions utilizing existing services within primary healthcare structure is limited. The PromeTheus trial aimed to evaluate the effectiveness of a home‐based, multifactorial, interdisciplinary intervention to prevent functional and mobility decline in (pre)frail older adults.

**Methods:**

In this multicentre, assessor‐blinded, randomized controlled trial, 385 community‐dwelling (pre)frail older adults (clinical frailty scale 4–6, ≥ 70 years) were randomly allocated (1:1) into the intervention group (IG: *n* = 196) or control group (CG: *n* = 189). The IG underwent the PromeTheus programme for 12 months, which included an obligatory unsupervised home‐based physical exercise programme and facultative counselling services (person‐environment fit, nutrition and coping with everyday life), implemented through existing healthcare services and referral to community group activities. The CG received usual care. The first primary outcome was the function component of the Late‐Life Function and Disability Instrument (LLFDI‐FC) after 12 months; Life‐Space Assessment (LSA) served as the second primary outcome. Secondary outcomes included participation (short‐form LLFDI disability component, LLFDI‐DC), frailty status, physical capacity (Short Physical Performance Battery, SPPB) and fall rate. Data analyses followed the intention‐to‐treat principle. An exploratory stratified analysis according to baseline physical capacity (SPPB ≤ 6 points [*n* = 210] vs. SPPB > 6 points [*n* = 175]) was also conducted.

**Results:**

Participants had a mean age of 81.2 ± 5.9 years, with 73.5% (*n* = 283) being female. At the 12‐month follow‐up, a significant between‐group difference in favour of the IG was observed for the change in the LLFDI‐FC (1.38 points, 95% confidence interval [CI] 0.08, 2.68) but not for the change in the LSA (0.49 points, 95% CI –3.65, 4.64). Change in frailty status (odds ratio for being in a ‘better’ change status 1.72, 95% CI 1.11, 2.64) and SPPB (0.58 points, 95% CI 0.10, 1.05) also showed significant between‐group differences in favour of the IG. The intervention did not affect the short form LLFDI‐DC or fall rate (*p* = 0.055–0.689). The stratified analysis showed significant improvements in the LLFDI‐FC, short‐form LLFDI‐DC (limitation), frailty status and SPPB (*p* = 0.002–0.020) in the IG compared to the CG for participants with SPPB ≤ 6 points but not for those with SPPB > 6 points. There were no study‐related serious adverse events.

**Conclusions:**

The PromeTheus programme had positive effects on physical functioning, frailty status and physical capacity but not on life‐space mobility and fall rate in community‐dwelling (pre)frail older adults. Participants with lower baseline physical capacity may benefit more from the programme.

## 1 Introduction

Frailty is a complex age‐related clinical condition characterized by decreased physiological reserves and reduced resistance to stressors, resulting from cumulative declines across multiple physiological systems [1]. Among community‐dwelling adults aged 70 years and older, frailty and prefrailty are common, with prevalence rates of 20% and 49%, respectively [2]. Older adults with (pre)frailty are vulnerable to numerous adverse health outcomes, such as functional and mobility disability, falls, hospitalization and mortality [1]. Consequently, the prevention and management of frailty have emerged as one of the most important public health challenges in ageing societies [S1].

In many industrialized nations, inpatient geriatric healthcare models for acute treatment and/or postacute rehabilitation are available when older adults experience acute medical events. However, in the absence of such events, physiotherapy prescribed by general practitioners (GPs) is often the only available community‐based service to prevent or mitigate functional decline. Typically one‐dimensional and episodic, this service is neither specifically tailored to the complex needs of (pre)frail older adults nor sustainable in the long term. Although inpatient services may offer certain benefits, they are costly from a health economic perspective and also often undesirable from an individual perspective. Many older adults prefer to remain in their homes and ‘age in place’ rather than be admitted to inpatient facilities [3]. Inpatient care can be associated with complications such as nosocomial infections [S2] and an increased risk of falls in unfamiliar environments [S3], further highlighting the need for community‐based alternatives. Geriatric medicine is inherently complex, multifactorial and interdisciplinary, also including preventive aspects [S4]. Transferring aspects of this comprehensive approach to the ambulatory setting could provide a promising strategy to improve physical functioning, reduce frailty and prevent disability in community‐dwelling (pre)frail older adults. However, evidence for complex frailty interventions initiated in primary care remains fragmented [4, 5].

An extensive body of evidence supports the effectiveness of physical exercise interventions in improving frailty, physical functioning and mobility among community‐dwelling (pre)frail older adults [6, 7] [S5]. Given these well‐documented benefits, physical exercise is strongly recommended for the prevention and management of frailty in evidence‐based practice guidelines [8]. These guidelines also emphasize the importance of targeting other major contributors to frailty. Malnutrition is a well‐known risk factor for frailty and often coincides with it [9]. Nutritional interventions, either alone or combined with physical exercise [6, 10], have been shown to reduce functional decline and frailty, with some evidence suggesting that the combination may be more effective than either approach alone [11]. Poor psychosocial resources, such as social isolation and loneliness, can also contribute to frailty progression [12] and account for the highest proportion of self‐perceived unmet care needs among frail older adults within primary care [13]. There is also some evidence that psychosocial interventions that enhance social capital may positively impact frailty status [14]. The home environment plays a critical role in maintaining independence in old age. Environmental modifications at home can improve physical functioning, enhance participation and prevent falls in community‐dwelling older adults [15].

Despite the availability of evidence‐based practice guidelines for frailty prevention and management [8], there is still a significant need for large‐scale, well‐designed randomized controlled trials (RCTs) that evaluate the effectiveness of frailty interventions implemented within and utilizing already existing healthcare resources, services and structures in the primary care setting [7]. Additionally, comprehensive preventive intervention programs for community‐dwelling (pre)frail older adults have not yet been implemented in the German healthcare system.

We developed the PromeTheus (‘prevention for more participation in old age’) programme, a 12‐month multifactorial, interdisciplinary intervention that includes an obligatory home‐based physical exercise programme delivered by physiotherapists (‘Weight‐bearing Exercise for Better Balance’ [WEBB] programme), along with facultative components comprising counselling services (on person‐environment fit, nutrition and/or coping with everyday life), implemented through existing resources and services of German healthcare providers, and referral to existing group programmes in the community [16]. The PromeTheus programme was based on the frailty intervention trial (FIT) programme, which was developed in Australia and demonstrated effectiveness in reducing frailty and improving physical capacity and mobility in community‐dwelling frail older persons who have been discharged from hospital and community rehabilitation services [17, 18]. In contrast, the PromeTheus programme targets (pre)frail older adults in the primary care setting, with the intention that primarily GPs screen and refer eligible individuals to the programme.

The primary aim of this RCT was to determine whether the home‐based, multifactorial, interdisciplinary PromeTheus programme prevents functional and mobility decline in community‐dwelling (pre)frail older adults compared to usual care. The secondary aim was to evaluate the effects of the PromeTheus programme on participation, frailty, physical capacity and falls.

## 2 Methods

### 2.1 Study Design

The PromeTheus study was a multicentre, assessor‐blinded RCT conducted in Heidelberg, Stuttgart and Ulm, located in Baden‐Wuerttemberg, Germany. Data were collected at baseline (T0), and 6 (T1) and 12 months (T2) after the baseline assessment (±2 weeks), with all assessments conducted at the participant's home. The study protocol was preregistered at the German Clinical Trials Register (DRKS00024638) on 11 March 2021 and has been published elsewhere [16]. There were no significant deviations from the protocol. The study was approved by the local ethics committees at each study site (Heidelberg: S‐072/2021; Stuttgart: 732/2020B01; Ulm: 26/21) and by the Ethics Committee of the State Medical Association Baden‐Wuerttemberg (B‐F‐2021‐042) and conducted in accordance with the 1964 Declaration of Helsinki and its later amendments. All participants provided written informed consent prior to study inclusion.

### 2.2 Recruitment and Eligibility Criteria

Eligible individuals were 70 years or older, prefrail, mildly frail or moderately frail (Clinical Frailty Scale [CFS] score = 4–6), living at home or in assisted living facilities, able to walk ≥ 10 m with or without a walking aid and members of the largest health insurance company in the German federal state of Baden‐Wuerttemberg (‘Allgemeine Ortskrankenkasse [AOK] Baden‐Württemberg’). Individuals who reported being able to walk ≥ 800 m without a walking aid or breaks or who had cognitive impairment (Short Orientation‐Memory‐Concentration Test [SOMCT] score > 10 points) were excluded. A detailed list of further exclusion criteria can be found in the study protocol [16].

Potential participants were recruited via GPs during routine visits (‘GP recruitment’) and flyers in AOK health magazines, articles in local newspapers and personalized letters sent out to potentially eligible AOK members (≥ 70 years, living at home or in assisted living facilities) (‘direct recruitment’). Individuals interested through direct recruitment could contact the local study centre for detailed study information and were referred to a GP for initial eligibility screening. In the case of positive GP screening, a telephone screening and home visit for cognitive screening were scheduled with the study centre to confirm further eligibility criteria. Further details on the recruitment strategies and screening process have already been published [16, 19].

### 2.3 Randomization and Blinding

Participants were randomly allocated (1:1 ratio) after the baseline assessment to the intervention group (IG) or control group (CG) through block randomization, stratified by study site. Randomization was conducted externally by the Institute of Epidemiology and Medical Biometry, Ulm University, Ulm, Germany. Participants were informed of their group assignment by physiotherapists. Assessors were blinded to the group allocation. Prior to the home assessments, participants were instructed to store any training materials out of sight to prevent assessors from becoming aware of their group assignment.

### 2.4 Intervention

The IG received the PromeTheus programme, which included the WEBB programme [18] as the obligatory core component, along with counselling services on person‐environment fit, nutrition and coping with everyday life as facultative individual components, and integration into exercise and/or social‐communicative group programmes in the community as a facultative group component.

The WEBB programme consisted of unsupervised, regular home‐based exercise. Participants were instructed to perform (a) ≥ 2 balance/coordination exercises (3 × 20 s for static or 3 × 10 repetitions for dynamic exercises), (b) ≥ 3 lower extremity strength exercises (2 × 10–15 repetitions; Borg 6–20 Rating of Perceived Exertion [RPE] = 15 [moderate‐to‐vigorous intensity]) on 3 days per week; and (c) 20‐ to 30‐min endurance sessions (e.g., walking, bicycling and swimming) at moderate intensity (‘noticeable exertion, but conversation still possible’) on 2–3 days per week.

Balance/coordination exercises included standing in various positions (e.g., semitandem, tandem and one‐legged stance), graded reaching tasks in standing (e.g., at different heights [floor, hip and overhead] and in different directions), stepping in multiple directions or over objects and walking practice (e.g., along a wall, sideways, tandem or over obstacles).

Strength exercises included sit‐to‐stands, heel raises, lateral and forward step‐ups and wall‐supported half squats. Individual tailoring and progression of balance/coordination exercises were based on reducing support (from with to without hand support, decreasing the base of support and standing on unstable surface), increasing the range or speed of movement (e.g., larger reaching movements and longer or faster steps) or adding a concurrent task (e.g., head movements and eyes closed). Strength exercises were tailored and progressed by lowering seat height (sit‐to‐stands), increasing range of motion (heel raises) or step height (step ups) or adding weight. Participants were instructed to perform the strength exercises briskly but in a controlled manner (concentric phase), to hold the end position for about 1 s and to return to the starting position slowly over about 2 s (eccentric phase). The prescribed strength‐training intensity for the strength exercises of approximately 15 on the Borg 6–20 RPE scale may correspond to about 60%–80% of one‐repetition maximum in older adults [S6–S8], although this relationship can be highly variable, particularly in frail populations and in unsupervised training settings.

The prescribed volume of endurance sessions was below the World Health Organization (WHO) recommendation of 150–300 min of moderate‐intensity aerobic physical activity (PA) per week but was intended to complement rather than replace participants' habitual PA. WHO recommendations refer to total weekly moderate‐intensity PA accumulated across domains of daily life, including work, leisure, home and transportation [S9].

Training materials included a balance pad, antislip mat, weight vest, training wedge, objects for stepping over or reaching towards (e.g., books and water bottle) and household items such as a table or chair (for hand support), cushion (for seat height adjustment) and stairs (for step‐ups). Two physiotherapists at each study side accompanied the WEBB programme through 10 home visits and five phone calls during the 12‐month intervention period. All physiotherapists were trained in implementing the WEBB programme prior to its delivery and followed a standardized trainer manual containing detailed information on the exercise content, procedures, progression and safety aspects. All participants in the IG received a German WEBB programme manual with information on general training principles, exercise descriptions and guidance on exercise progression, variation and safety precautions. They also received a workbook with training sheets containing their current exercise recommendations and prescriptions.

The need and willingness for the facultative components were assessed by physiotherapists during home visits using standardized checklists and screening tools for person‐environment fit (home environment and assistive devices), coping with everyday life (self‐management and psychosocial support) and nutritional status (malnutrition and anorexia), as well as a semistructured interview for potential interest and referral to local group programmes (physical activities, social‐communicative, musical or creative‐artistic). If one of these assessments was positive, participants received (a) recommendations for environmental adaptations and information on local healthcare supply stores and municipal/nonprofit counselling centres, or for discussing assistive device prescriptions with their GP (→ person‐environment fit); (b) referral to a social worker from the AOK health insurance for health and social case management (→ coping with everyday life); (c) referral to a nutritionist from the AOK health insurance for counselling on prevention and treatment strategies for malnutrition (→ nutritional status); or (d) information about local counselling sites on group activities for older people (→ group activities). A systematic description of the PromeTheus programme is provided in the study protocol [16].

### 2.5 Control

The CG received usual care, consisting of standard health and aged care services routinely available to older people in the German healthcare system. These include consultations with GPs and medical specialists, nursing care and allied health services such as prescriptions for physiotherapy when medically indicated. The German statutory health and long‐term care insurance systems provide broad access to healthcare services. Aged care services are covered by the long‐term care insurance based on assessed care levels. Depending on their needs, older adults may receive cash benefits or professional home care services. Beyond that, the CG received a leaflet on global recommendations for PA and nutrition in old age and discussed these aspects with a physiotherapist during a one‐time, 45‐min home visit.

### 2.6 Primary Outcomes

The first primary outcome was the function component of the Late‐Life Function and Disability Instrument (LLFDI) [20]. The LLFDI function component is a well‐established patient‐reported outcome measure (PROM) to assess physical functioning in daily life among community‐dwelling older adults (scoring range = 0–100 points; higher scores indicate higher physical functioning). The construct validity, test–retest reliability and sensitivity to change of the LLFDI function component have been documented in previous studies [20] [S9].

The University of Alabama at Birmingham Life‐Space Assessment (LSA) [21] served as the second primary outcome. It assesses self‐reported life‐space mobility, considering the extent, frequency and independence of mobility in the previous 4 weeks (scoring range = 0–120 points; higher scores indicate greater life‐space mobility). Feasibility, construct validity, test–retest reliability and sensitivity to change of the LSA have been established previously [22].

### 2.7 Secondary Outcomes

Secondary outcomes included participation (short form LLFDI [SF‐LLFDI] disability component), frailty status, physical capacity (Short Physical Performance Battery, SPPB) and fall rate. Frailty status was assessed according to the five criteria of the Fried frailty phenotype [1]: (1) self‐reported unintentional weight loss (≥ 1 kg in the last 3 months); (2) exhaustion (two items from the Center for Epidemiological Studies Depression Scale); (3) low PA (< 150 min of moderate‐to‐vigorous PA per week following the WHO recommendations, measured with the German Physical Activity Questionnaire 50+); (4) slowness (gender‐ and height‐adjusted slow gait speed); and (5) weakness (gender‐adjusted and body mass index (BMI)–adjusted low handgrip strength). Participants were categorized into three groups: robust (0 criteria), prefrail (1–2 criteria) and frail (≥ 3 criteria) [1]. The SPPB comprises three tests of lower extremity function: a hierarchical standing balance test (side‐by‐side, semitandem and tandem stance), a usual gait speed test over 4 m and a 5‐chair stand test. Each subtest is scored from 0 to 4 points, yielding a total score ranging from 0 to 12, with higher scores indicating better physical capacity [S11]. The fall rate was derived from fall calendars completed by participants for each week over the 12‐month study period and returned quarterly to the study site. Training adherence to the WEBB programme was assessed by self‐report via the Exercise Adherence Rating Scale (EARS, 0–24 points) at Home Visits 4, 7 and 9. A mean EARS score (calculated across the three home visits) of ≥ 17 points was considered acceptable adherence [23]. In addition, the physiotherapist primarily responsible for each participant estimated a global level of training adherence categorized into five levels (0%, ≤ 25%, 26%–50%, 51%–75% and 76%–100%) [17].

### 2.8 Descriptive Measures

Age, sex, BMI, CFS, gait speed (derived from the SPPB 4‐m gait speed test), SOMCT and self‐reported fall history in the last 6 months were collected to describe participant characteristics.

### 2.9 Safety

Adverse events were monitored by all study staff as they occurred, and during the intervention‐related home visits and telephone calls, and home assessments. Serious adverse events were defined as any harmful condition or injury that resulted in hospitalization, death or caused persistent disability or incapacity.

### 2.10 Sample Size

An a priori sample size calculation was performed for a group comparison at 12 months in the LLFDI function component, based on baseline data from the LiFE (‘Lifestyle integrated Functional Exercise’) study [24] and the substantial meaningful change reported for the LLFDI function component [25], resulting in a sample size of 200 participants per group. Further information on this calculation is provided in the study protocol [16].

### 2.11 Statistical Analysis

**FIGURE 1:**
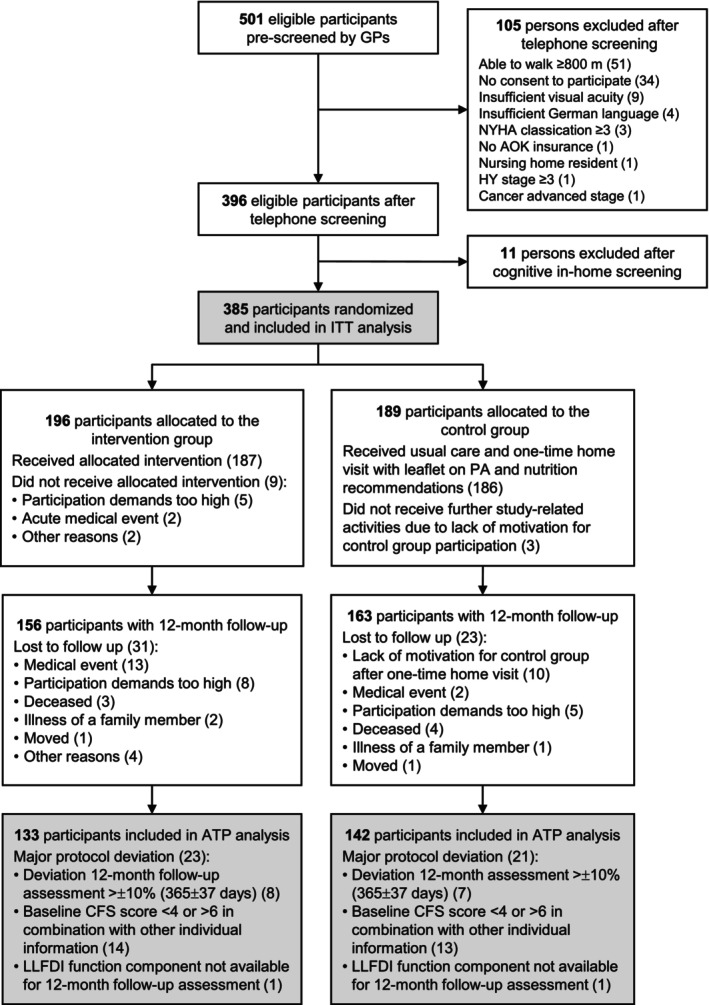
Flowchart of the participants in the study. AOK, Allgemeine Ortskrankenkasse; ATP, according‐to‐protocol; CFS, Clinical Frailty Scale; GP, general practitioner; HY, Hoehn & Yahr; ITT, intention‐to‐treat; LLFDI, Late‐Life Function and Disability Instrument; NYHA, New York Heart Association; PA, physical activity.

Baseline characteristics of the study population are reported as absolute and relative frequencies for categorical variables, medians with first and third quartiles for skewed continuous variables and arithmetic means with standard deviations (SD) for other continuous variables. All analyses were performed using R version 4.3.2 (R Foundation for Statistical Computing, Vienna, Austria). Primary statistical analyses followed an intention‐to‐treat (ITT) approach, including all randomized participants. Multiple imputation for longitudinal data by chained equations was conducted using the mice function (mice package) [S12]. Variables included in the multiple imputation model were selected based on clinical relevance and included participant characteristics, design variables (treatment arm and study site) and primary and secondary outcome measures collected at all time points (T0, T1 and T2). Primary outcomes were also analysed in an according‐to‐protocol (ATP) analysis, which included participants who adhered to the assigned treatment regimen until the 12‐month follow‐up (Figure 1). Group differences in primary outcomes were tested using two‐sample *t* tests. A two‐sided *p* value of less than 0.05 indicated statistical significance. No corrections for multiplicity were made for the two primary outcomes, because a fixed sequence testing procedure was applied with hypotheses tested in a prespecified order (the second primary outcome was tested only if the first was significant at *p* < 0.05). A linear mixed‐effects model (LMM), fitted with the lmer function (lme4 package), was applied to estimate the difference in expected arithmetic means with 95% confidence intervals (CI) for continuous dependent variables. Changes in frailty status from baseline to 12‐month follow‐up were assessed as an outcome variable with three ordered categories (deterioration, no change and improvement) using an ordered logistic regression model fitted with the polr function (MASS package; the proportional odds assumption was not violated). A generalized linear model (GLM) with a Gaussian distribution and log link function was used to assess the association between the study arm and the frequency of falls by estimating fall rate ratios (95% CI) based on imputed fall rates, fitted with the glm function (stats package). Additionally, exploratory stratified analyses of the primary and secondary outcomes were conducted according to the physical capacity at baseline, defined as low (SPPB ≤ 6 points) and high (SPPB > 6 points) [S11]. Risk and rate ratios with 95% CI were estimated using Quasi‐Poisson regression models to compare the probability of experiencing at least one (serious) adverse event and the incidence rates (events per person‐year) of (serious) adverse events between groups.

## 3 Results

### 3.1 Participant Flow and Baseline Characteristics

Participants were recruited from May 2021 to November 2022 with the first enrolled on 25 May 2021 and the last completing the study on 27 November 2023. Details on screening and recruitment strategies have been reported elsewhere [19]. Out of 501 potential participants pre‐screened by GPs, 385 were eligible after telephone and cognitive in‐home screening and were randomized into the IG (*n* = 196) and CG (*n* = 189). Baseline characteristics were well‐balanced between the two groups (Table 1). Twelve (3.1%) participants dropped out directly after randomization and did not receive any further study‐related measures during the intervention period (IG: *n* = 9, 4.6%, CG: *n* = 3, 1.6%). Another 54 were lost to the 12‐month follow‐up (IG: *n* = 31, 15.8%, CG: *n* = 23, 12.2%). Figure 1 shows the flow of participants through the study.

**TABLE 1:** Baseline characteristics of the study population.

Variable	IG (*n* = 196)	CG (*n* = 189)
Age (years), mean (SD)	81.7 (5.9)	80.7 (5.8)
Female, *n* (%)	146 (74.5)	137 (72.5)
BMI (kg/m^2^), mean (SD)	29.1 (6.1)	29.8 (5.5)
Clinical Frailty Scale score, median [Q1, Q3]	4 [4, 5]	4 [4, 5]
Frailty status, *n* (%)		
Robust	28 (14.3)	23 (12.2)
Prefrail	88 (44.9)	93 (49.2)
Frail	71 (36.2)	70 (37.0)
NA	9 (4.6)	3 (1.6)
Gait speed (m/s), mean (SD)	0.68 (0.24)	0.67 (0.23)
SOMCT score, median [Q1, Q3]	4 [2, 8]	4 [2, 6]
Fall within last 6 months, *n* (%)	78 (39.8)	64 (33.9)
LLFDI function component score, mean (SD)	47.2 (7.6)	47.9 (7.8)
SF‐LLFDI disability component score, mean (SD)		
Frequency	24.9 (4.5)	25.5 (4.3)
Limitation	28.6 (7.5)	30.4 (6.2)
Life‐Space Assessment score, mean (SD)	48.3 (19.7)	49.8 (17.7)
SPPB score, mean (SD)	6.4 (2.8)	6.5 (2.6)
SPPB score ≤ 6 pts., *n* (%)	110 (56.1)	100 (52.9)

Abbreviations: BMI, Body Mass Index; LLFDI, Late‐Life Function and Disability Instrument; NA, not available; Q1, first quartile; Q3, third quartile; SD, standard deviation; SF‐LLFDI, Short‐Form Late‐Life Function and Disability Instrument; SOMCT, Short Orientation‐Memory‐Concentration Test; SPPB, Short Physical Performance Battery.

The total sample had a mean age of 81.2 ± 5.9 years, and 73.5% (*n* = 283) were female. About half (*n* = 181, 47.0%) were categorized as prefrail, 36.6% (*n* = 141) as frail and 13.2% (*n* = 51) as robust, according to the Fried frailty criteria. More than one third (*n* = 142, 36.9%) reported a fall history within the last 6 months. Physical capacity was generally limited, with a mean SPPB score of 6.5 ± 2.7 points. In total, 210 participants (54.5%) had a SPPB score ≤ 6 points, and 175 participants (45.5%) had a SPPB score of > 6 points.

### 3.2 Intervention Dose and Adherence

During the intervention period, the IG received an average of 8.6 ± 2.9 home visits and 4.1 ± 1.7 telephone calls from the physiotherapists.

Self‐reported training adherence to the obligatory home‐based exercise programme was acceptable (EARS ≥ 17 points) for 76% of participants in the IG. The mean EARS score across the three home visits was 19.4 ± 3.7 points, also indicating an overall high training adherence. EARS scores showed a slight decrease over time, with 20.3 ± 4.1 points at Visit 4, 19.5 ± 4.5 points at Visit 7 and 19.1 ± 5.4 points at Visit 9 (*n* = 138 with complete EARS data). The median global adherence level rated by the physiotherapists was in the category of 51% to 75%, with about one third (30.6%) having a high training adherence of 76%–100% (Table 2).

**TABLE 2:** Adherence to the obligatory home‐based exercise programme and participation in the facultative components of the PromeTheus programme.

Intervention components	*n* = 196^a^
Training adherence to home‐based exercise programme (WEBB)	
EARS (participant rating) (*n* = 175)^b^, ^c^	
Mean (SD)	19.4 (3.7)
≥ 17 pts., *n* (%)	133 (76.0)
Global adherence level (physiotherapist rating), *n* (%)	
76%–100%	60 (30.6)
51%–75%	47 (24.0)
26%–50%	37 (18.9)
15%–25%	31 (15.8)
0%	21 (10.7)
Counselling on person‐environment fit	
No identified need, *n* (%)	133 (67.9)
Identified need and received recommendations, *n* (%)	45 (23.0)
Identified need but unwilling to receive recommendations, *n* (%)	1 (0.5)
Dropout before screening, *n* (%)	17 (8.7)
Counselling on coping with everyday life	
No identified need, *n* (%)	92 (46.9)
Identified need and received counselling, *n* (%)	46 (23.5)
Identified need but unwilling to receive counselling, *n* (%)	36 (18.4)
Dropout before screening, *n* (%)	22 (11.2)
Nutritional counselling	
No identified need, *n* (%)	117 (59.7)
Identified need and received counselling, *n* (%)	9 (4.6)
Identified need but unwilling to receive counselling, *n* (%)	47 (24.0)
Dropout before screening, *n* (%)	23 (11.7)
Group activities	
No interest, *n* (%)	94 (48.0)
Interest and received information, with referral, *n* (%)	28 (14.3)
Interest and received information, but no referral, *n* (%)	49 (25.0)
Dropout before interview, *n* (%)	25 (12.8)

Abbreviations: EARS, Exercise Adherence Rating Scale; WEBB, Weight‐bearing Exercise for Better Balance.

^a^ Unless otherwise indicated.

^b^ Unavailable in 21 participants due to dropout before first EARS assessment at Home Visit 4.

^c^ Based on mean values calculated for the EARS assessments performed at Home Visits 4, 7 and 9.

The rates of identified needs for the facultative counselling services of the PromeTheus programme ranged from 23.5% to 41.8%, with the highest rate for coping with everyday life (Table 2). Actual receipt of these services, based on participants' willingness, ranged from 4.6% to 23.5%. Interest in group activities was expressed by 39.3% of participants, resulting in referrals for 14.3% in total.

### 3.3 Primary Outcomes

**FIGURE 2:**
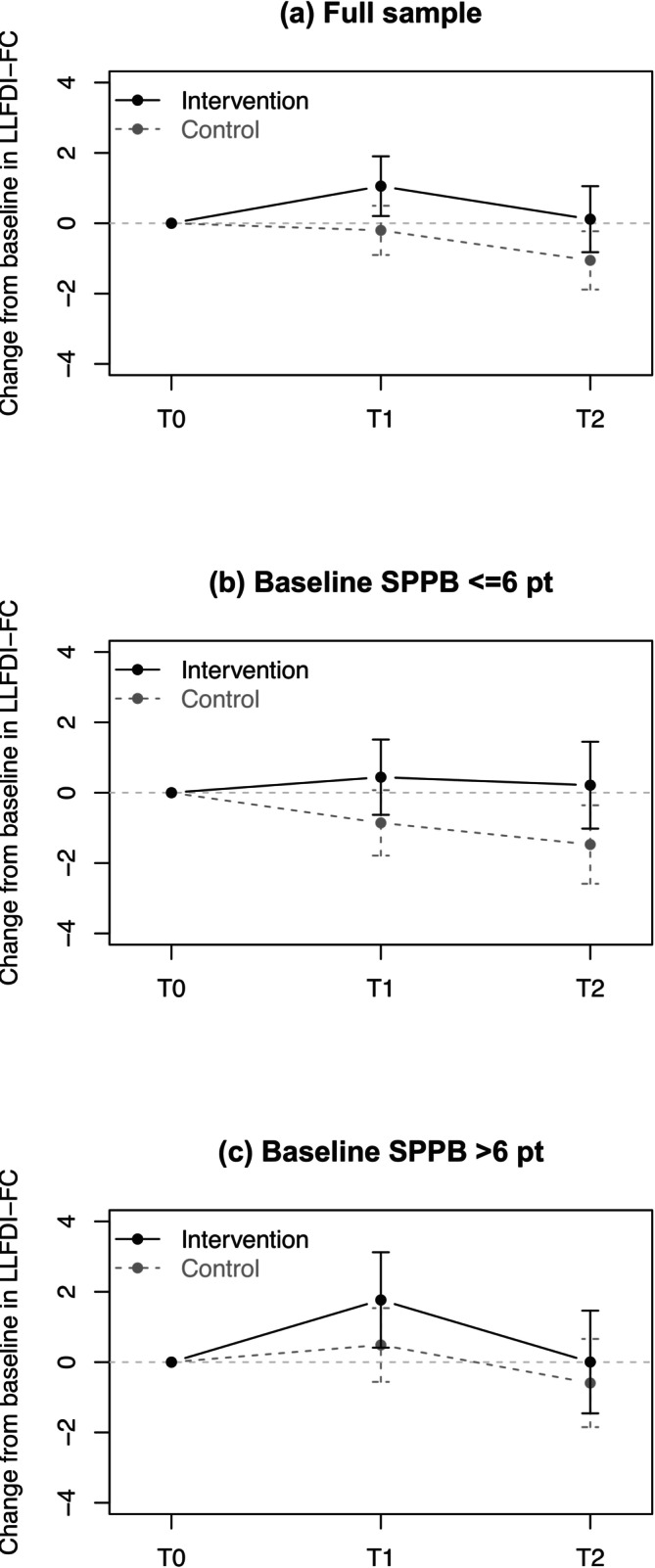
Change in Late‐Life Function and Disability Instrument function component (LLFDI‐FC) from baseline (T0) to 6‐month follow‐up (T1) and 12‐month follow‐up. Plots show the mean with 95% confidence intervals. Solid dark line = intervention group; dashed dark grey line = control group. Plots are given for (a) the total sample and stratified groups with (b) low (Short Physical Performance Battery [SPPB] ≤ 6 points) and (c) high (SPPB > 6 points) baseline physical capacity.

A between‐group difference of 1.38 points in favour of the IG was observed for the change in the LLFDI function component after 12 months (T2) showing statistical significance using the two‐sample *t*‐test (*p* = 0.038; Table 3 and Figure 2). This was confirmed by a LLM considering all three time points (1.19 points, 95% CI 0.08, 2.31) and even more pronounced when an ATP analysis was performed (two‐sample *t* test: 1.55 points, *p* = 0.021; LMM: 1.55 points, 95% CI 0.36, 2.74). The intervention showed no significant effect on the LSA, the second primary outcome, irrespective of the analytical approach (*p* = 0.815–0.989).

**TABLE 3:** Effect of the PromeTheus programme after 12 months on primary and secondary outcomes.

Outcome	Analysis	Model/statistical test	Estimate (95% CI)	*p* value
Primary outcomes				
LLFDI function component (first)	ITT	Multiple imputation two sample *t* test^a^	1.38 (0.08, 2.68)	0.038
	ITT	Linear mixed‐effects model^a^	1.19 (0.08, 2.31)	0.037
	ATP	Two sample *t* test^a^	1.55 (0.23, 2.87)	0.021
	ATP	Linear mixed‐effects model^a^	1.55 (0.36, 2.74)	0.011
Life‐space assessment (second)	ITT	Multiple imputation two sample *t* test^a^	0.49 (−3.65, 4.64)	0.815
	ITT	Linear mixed‐effects model^a^	0.23 (−3.47, 3.94)	0.902
	ATP	Two sample *t* test^a^	0.03 (−4.31, 4.37)	0.989
	ATP	Linear mixed‐effects model^a^	−0.21 (−4.23, 3.82)	0.921
Secondary outcomes				
SF‐LLFDI disability component				
Frequency	ITT	Linear mixed‐effects model^a^	0.14 (−0.53, 0.80)	0.689
Limitation	ITT	Linear mixed‐effects model^a^	1.66 (−0.03, 3.34)	0.055
Frailty status	ITT	Multiple imputation ordinal logistic regression model^b^	1.72 (1.11, 2.64)	0.014
SPPB	ITT	Linear mixed‐effects model^a^	0.58 (0.10, 1.05)	0.017
Fall rate	ITT	Multiple imputation generalized linear model^c^	0.99 (0.70, 1.39)	0.947

Abbreviations: ATP, according‐to‐protocol (*n* = 275); CI, confidence interval; ITT, intention‐to‐treat (*n* = 385); LLFDI, Late‐Life Function and Disability Instrument; SF‐LLFDI, Short‐Form Late‐Life Function and Disability Instrument; SPPB, Short Physical Performance Battery.

^a^ Estimate is the difference in means (change between baseline and 12 months) between the intervention group and the control group.

^b^ Estimate is the proportional odds ratio of improvement versus no change or no change versus deterioration after 12 months in the intervention group compared to the control group.

^c^ Estimate is the fall rate ratio after 12 months between the intervention group over the control group.

The intervention effects on primary and secondary outcomes at the 6‐month follow‐up (T1) are provided in Table S1. Findings were similar to those observed at 12 months, with significant intervention effects on the LLFDI function component and SPPB, and no significant effects on the LSA. The beneficial effect on frailty status observed at 12 months was not present at 6 months.

### 3.4 Secondary Outcomes

**FIGURE 3:**
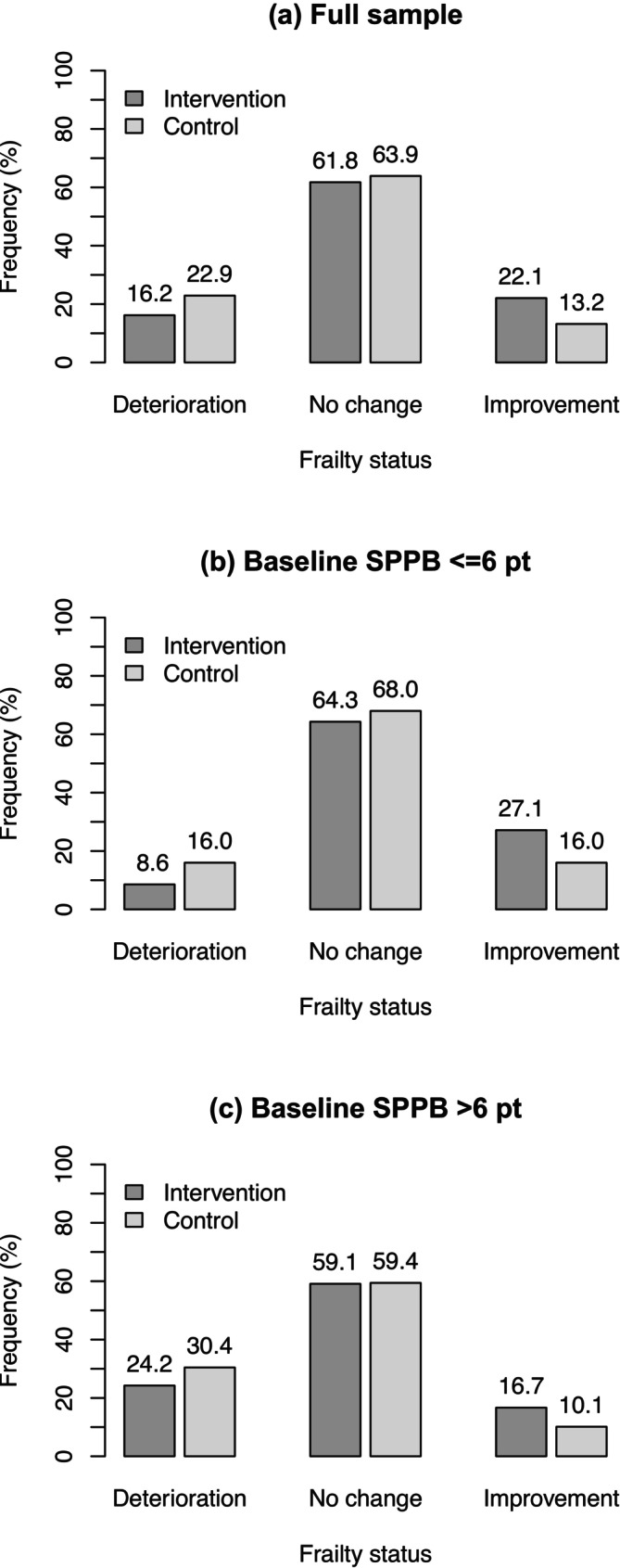
Frequency of deterioration, no change and improvement in frailty status from baseline to 12‐month follow‐up. Bar plots are given for (a) the total sample and stratified groups with (b) low (Short Physical Performance Battery [SPPB] ≤ 6 points) and (c) high (SPPB > 6 points) baseline physical capacity. Dark grey bars = intervention group; light grey bars = control group.

A positive intervention effect after 12 months was observed on frailty status, with the odds ratio for improvement versus no change and no change versus deterioration, respectively, of 1.72 times higher in the IG than in the CG (proportional odds ratio [OR] 1.72, 95% CI 1.11, 2.64) (Table 3 and Figure 3). In addition, the SPPB was positively influenced by the intervention (0.58 points, 95% CI 0.10–1.05). The intervention did not significantly affect the SF‐LLFDI disability component (frequency dimension: 0.14 points, 95% CI −0.53, 0.80; limitation dimension: 1.66 points, 95% CI −0.03, 3.34) or the fall rate (ratio: 0.99, 95% CI 0.70, 1.39).

### 3.5 Outcomes Stratified by Baseline Physical Capacity

Participants with lower physical capacity at baseline (SPPB ≤ 6 points) benefited particularly from the PromeTheus programme, with significant improvements in the LLFDI function component (1.72 points, 95% CI 0.32, 3.13), SF‐LLFDI disability component (limitation dimension: 3.20 points, 95% CI 0.52, 5.89), frailty status (OR 2.05, 95% CI 1.10, 3.82) and SPPB (1.01 points, 95% CI 0.39, 1.63) (Table 4).

**TABLE 4:** Effect of the PromeTheus programme after 12 months on primary and secondary outcomes stratified by low (≤ 6 points) versus high (> 6 points) baseline Short Physical Performance Battery (SPPB).

Outcome	Analysis	SPPB	Estimate (95% CI)	*p* value
Primary outcomes				
LLFDI function component (first)	ITT^a^	Low	1.72 (0.32, 3.13)	0.017
		High	0.57 (−1.17, 2.31)	0.525
	ATP^a^	Low	2.32 (0.89, 3.76)	0.002
		High	0.63 (−1.32, 2.58)	0.529
Life‐Space Assessment (second)	ITT^a^	Low	2.14 (−2.54, 6.83)	0.372
		High	−2.02 (−7.75, 3.71)	0.491
	ATP^a^	Low	1.95 (−2.95, 6.84)	0.438
		High	−2.72 (−9.31, 3.88)	0.422
Secondary outcomes				
SF‐LLFDI disability component				
Frequency	ITT^a^	Low	0.01 (−0.96, 0.99)	0.983
		High	0.26 (−0.64, 1.16)	0.575
Limitation	ITT^a^	Low	3.20 (0.52, 5.89)	0.020
		High	−0.29 (−2.11, 1.54)	0.758
Frailty status	ITT^b^	Low	2.05 (1.10, 3.82)	0.025
		High	1.40 (0.76, 2.56)	0.279
SPPB	ITT^a^	Low	1.01 (0.39, 1.63)	0.002
		High	0.05 (−0.64, 0.73)	0.894
Fall rate	ITT^c^	Low	1.09 (0.72, 1.64)	0.683
		High	0.65 (0.38, 1.11)	0.116

Abbreviations: ATP, according‐to‐protocol (*n* = 275); CI, confidence interval; ITT, intention‐to‐treat (*n* = 385); LLFDI, Late‐Life Function and Disability Instrument; SF‐LLFDI, Short‐Form Late‐Life Function and Disability Instrument.

^a^ Linear mixed‐effects model with estimates given for the difference in the expected arithmetic means (change between baseline and 12 months) between the intervention group and the control group.

^b^ Multiple imputation ordered logistic regression model with estimates given for the proportional odds ratio of improvement versus no change or no change versus deterioration after 12 months in the intervention group compared to the control group.

^c^ Multiple imputation generalized linear model with estimates given for the fall rate ratio after 12 months between the intervention group over the control group.

### 3.6 Safety

In the IG, 137 (69.9%) participants experienced at least one adverse event during the study, compared to 105 (55.6%) participants in the CG (risk ratio 1.26, 95% CI 1.08, 1.47). Serious adverse events occurred in 66 (33.7%) participants in the IG and 34 (18.0%) in the CG (risk ratio 1.60, 95% CI 1.19, 2.14). Adverse events occurred at a rate of 2.41 events per person‐year in the IG and 1.47 in the CG (rate ratio 1.65, 95% CI 1.27, 2.13) and serious adverse events at rates of 0.57 and 0.26 events per person‐year, respectively (rate ratio 2.23, 95% CI: 1.44–3.45) (Table S2). Four study‐related adverse events were reported that all occurred while performing the home‐based exercise programme and had only mild consequences: two participants experienced knee pain, and two participants experienced a noninjurious fall without any consequences. No study‐related serious adverse events were documented during the 12‐month study period.

## 4 Discussion

This multicentre randomized controlled trial investigated the effectiveness of the home‐based, multifactorial, interdisciplinary PromeTheus programme, implemented through existing healthcare structures and services, to prevent functional and mobility decline in community‐dwelling (pre)frail older adults. After 12 months, positive intervention effects were observed on the LLFDI function component, the first primary outcome, but not on the LSA, the second primary outcome. The intervention also positively impacted secondary outcomes, including frailty status and physical capacity (SPPB).

The effect on the LLFDI function component reached a small meaningful change—defined as a 2‐point difference (rounded to the nearest whole number) [25]—in the ATP analysis of the total sample (1.55 points), and both the ITT (1.72 points) and ATP (2.32 points) analyses among the subgroup of participants with low baseline physical capacity.

The beneficial effect on self‐reported physical functioning in daily life (LLFDI) contrasts with findings reported for the FIT programme in Australia, from which the PromeTheus programme was derived. The FIT programme showed no beneficial effect on PROMs of daily‐life functioning (Reintegration to Normal Living Index, Nottingham Extended Activities of Daily Living Index), though it led to improvements in frailty status and physical capacity [17, 18]. Previous studies have also reported mixed results regarding the effectiveness of other home‐based preventive programmes with unsupervised physical exercise as the core component, supported by physiotherapists, in community‐dwelling frail older adults [26, 27].

Although an improvement in life‐space mobility was reported for the FIT programme [18], no such effect was observed in the present study. This discrepancy may be explained by COVID‐19‐related personal and environmental factors that restricted the potential for specifically addressing and increasing the spatial extent of daily movement, a key determinant of life‐space mobility. Concerns about infections, social distancing and limited access to public services and group activities during the PromeTheus trial likely contributed to these findings. Another potential reason relates to differences in the settings and participant characteristics. The FIT programme targeted frail older adults discharged from hospital and community rehabilitation services, with substantially lower baseline life‐space mobility (LSA: IG = 27.6 ± 12.9 points, CG = 30.0 ± 14.3 points) compared to our participants (LSA: IG = 48.3 ± 19.7 points, CG = 49.8 ± 17.7 points). A lower initial level of life‐space mobility provides greater potential for improvement. Furthermore, older adults who have experienced a recent health event, such as hospitalization, may be more receptive, motivated and responsive to health interventions (‘teachable moment’) [S13], whereas modifying habitual behaviour may be more challenging in older adults without such an event.

Participants receiving the PromeTheus programme had a 72% higher odds of being in a better category in frailty status (no change vs. deterioration or improvement vs. no change) after the intervention period compared to those receiving usual care. In addition, the beneficial effect on the SPPB in the total sample (0.58 points) and in the subgroup of participants with low physical capacity (1.01 points) exceeded the small (0.5 points) and substantial meaningful change (1.0 points), respectively [28]. These findings represent one of the most clinically relevant outcomes and align with those reported for the FIT programme. The FIT programme effectively reduced frailty prevalence (−15%) and improved physical capacity by a substantial meaningful change (SPPB +1.44 points) after 12 months among community‐dwelling frail older adults following hospital discharge and community rehabilitation services compared to usual care [17]. Even more, our findings extend this evidence by showing that similar benefits can be achieved for community‐dwelling (pre)frail older adults in primary care settings, broadening the applicability of such home‐based interventions. The smaller effect on the SPPB observed in our sample may be explained by the higher baseline physical capacity of our participants (SPPB: IG = 6.4 ± 2.8 points; CG = 6.5 ± 2.6 points) compared to those in the FIT (SPPB: IG = 5.2 ± 1.9 points; CG = 5.7 ± 2.1 points). According to the general dose–response relationship for exercise, individuals with higher physical capacity tend to experience smaller gains from similar training load. A higher intensity or dosage of the unsupervised exercises may have been required for some of our participants with higher baseline physical capacity to achieve larger improvements. Our findings in participants with lower baseline physical capacity (SPPB ≤ 6 points) support this suggestion, as this subgroup showed more pronounced and substantial improvements in the IG compared to the CG for the SPPB (+1.01 points), indicating that the home‐based exercise programme may be more suitable for individuals with lower physical capacity. Future implementations of the WEBB programme may consider strategies to optimize intensity, volume and progression, while maintaining feasibility and safety in the home‐based setting, to further enhance training effects, particularly for participants with higher physical capacity.

Overall, we observed that the intervention effects on the LLFDI function component, SF‐LLFDI disability component (limitation dimension)—a parameter that serves as a surrogate for participation [S14]—as well as frailty and SPPB were consistently larger in participants with lower baseline physical capacity. This finding aligns with other studies, such as the SPRINTT (Sarcopenia and Physical Frailty IN older people: multicomponenT Treatment) trial [29] or the LIFE (Lifestyle Interventions and Independence for Elders) study [30], which suggest that long‐term interventions with physical exercise as the core component, including also home‐based exercises, may be more effective in preventing the physical decline in (frail) older adults with lower physical capacity.

We did not observe an effect of the PromeTheus programme on fall rates, similar to the findings from the FIT programme, which did not result in lower fall rates after the 12 months compared to usual care in community‐dwelling frail older adults [31]. Additionally, previous RCTs also showed no benefits of the WEBB programme on 12‐month fall rates in older adults after a fall‐related leg or pelvic fractures [32] or a recent hospital stay, in which even an increase in fall rates was observed [33], despite its beneficial effects on physical capacity. These findings differ from previous evidence showing fall prevention effects of home‐based exercise in community‐dwelling older adults [34]. However, studies that have demonstrated such effects primarily included participants who were less frail than those in the PromeTheus study [24] [S15, S16]. Exercise interventions conducted in higher risk older populations tend to show smaller fall prevention effects [35], and evidence on fall prevention interventions in frail older adults remains inconclusive [36]. Indeed, our stratified analysis showed a nonsignificant trend towards reduced falls in participants with higher baseline physical capacity (fall rate ratio 0.65, 95% CI 0.38, 1.11). An exercise intervention with higher supervision levels (e.g., to increase adherence and monitor exercise intensity) or a more personalized multidomain intervention specifically targeting fall risk factors, as recommended for high‐risk and/or frail older adults [37], may be more effective for fall prevention than the PromeTheus programme, which primarily targeted the prevention of functional decline. Additionally, our study was not powered to detect differences in fall rates.

Home‐based exercise programmes often show poor adherence among older adults [38]. Findings from this study indicated, however, moderate to good self‐reported and physiotherapist‐rated adherence to the WEBB programme. Providing moderate levels of home visit support, physiotherapist guidance and telephone contacts may have contributed to this, as these characteristics have been identified as beneficial for enhancing adherence to home‐based exercise programmes [38]. The physiotherapists' rating of global adherence (51%–75%) was higher than that reported in the FIT study (25%–50%), which may be due to the inclusion of only frail older adults with lower physical capacity and multiple medical conditions [17, 18]. Better physical health and fewer comorbidities have been positively associated with higher adherence to home‐based exercise programmes [S17]. Additionally, findings from the mean EARS score also suggest good adherence, which was higher than reported for other home‐based training programmes with fewer personal and telephone contacts during the intervention period [39].

A key feature of the PromeTheus programme is that the obligatory home‐based exercise component was performed unsupervised by participants between physiotherapy contacts (home visits and telephone calls). Although this low‐level supervision format supports implementation in routine care by enhancing feasibility and reducing resource requirements, it may have limited the optimization and monitoring of exercise intensity, volume and progression and thereby influenced the magnitude of the observed effects, as higher levels of supervision in exercise interventions may provide additional benefits in older adults [S18].

A strength of the PromeTheus programme is that its home‐based exercise component relies on widely available existing structures of the German healthcare system, requiring fewer resources than, for example, outpatient rehabilitation. It proved to be feasible, safe and effective and was successfully piloted by three physiotherapy practices during the study period. However, a higher rate of (serious) adverse events was observed among participants receiving the PromeTheus programme compared to those receiving usual care. Higher rates of (serious) adverse events in exercise interventions compared to nonexercising controls have also been reported in the SPRINTT [29], LIFE [30] and FINGER (Finnish Geriatric Intervention Study to Prevent Cognitive Impairment) trials [40]. The more frequent contact between IG participants and study staff likely contributed to increased recognition and reporting of adverse events. Importantly, the number of intervention‐related adverse events was very low, and all were nonserious. Taken together, we believe this core component is highly transferable to routine care, as well as other countries and healthcare systems. However, the facultative counselling services on coping with everyday life and nutrition were delivered by participants' health insurance providers, which may be specific to the local healthcare system, potentially limiting the generalizability of the multifactorial, interdisciplinary intervention approach.

This study has several limitations. Intervention effects were mixed across the self‐reported primary outcomes, with benefits observed for the LLFDI function component but not for the LSA. Both outcomes were self‐reported and may therefore be subject to social desirability and/or reporting bias. In addition, the LSA may also be influenced by contextual factors not fully addressed by the intervention (e.g., seasonal conditions and transportation access).

The predominantly unsupervised nature of the home‐based exercise programme and the use of RPE‐based rather than objective load‐based intensity prescription may have increased variability in the delivered training stimulus and may have attenuated effects.

The primary recruitment strategy was intended to be referrals from GPs, with an alternative ‘direct recruitment’ approach—using flyers, newspaper articles and personalized letters—a priori defined as a secondary strategy. However, due to the limited capacity of GPs to screen or refer participants during the COVID‐19 pandemic (2021–2022), 84% of participants (*n =* 325) were ultimately recruited through the alternative ‘direct recruitment’ strategy. These participants had higher physical capacity (SPPB), life‐space mobility (LSA), and participation (SF‐LLFDI disability component) and were less frail compared to those recruited via GPs [19]. Consequently, the intervention effect may have been even greater if more participants had been referred by GPs.

The PromeTheus programme provides a multifactorial, interdisciplinary intervention with an unsupervised home‐based exercise programme as the obligatory core component, alongside additional facultative components. The identified need for these facultative components was lower than a priori expected, likely due to the previously mentioned adaptations in the recruitment strategy. Participants who were included on their own initiative may have already been aware of resources related to person‐environment fit, coping with everyday life or nutrition. Additionally, the provision of information on local group activities was severely limited due to the COVID‐19 pandemic, and referrals to such activities were virtually impossible at this time. As a result of the limited uptake of facultative components, the observed effect of the PromeTheus programme is likely primarily attributable to the obligatory home‐based exercise component.

The COVID‐19 pandemic significantly impacted the recruitment process, resulting in a final sample size of only 385 participants (96.3% of the a priori calculated target of 400), even after implementing the secondary recruitment strategy. Nonetheless, the study had sufficient power to detect a significant between‐group difference in the primary outcome.

Adherence to the home‐based exercise programme was also intended to be monitored using a 12‐month training diary provided to IG participants. However, it became evident that these diaries were in most cases completed inconsistently, only partially or not returned at all. Consequently, an analysis of training adherence based on the diaries was not feasible, and adherence and dose received could only be assessed indirectly using the self‐reported EARS and physiotherapist‐rated global adherence.

Findings from the stratified analyses by baseline physical capacity were exploratory and should be interpreted cautiously.

In conclusion, the home‐based multifactorial, interdisciplinary PromeTheus programme, which included an unsupervised exercise programme as its obligatory core component, had positive effects on physical functioning, frailty status and physical capacity but not on life‐space mobility and fall rate in community‐dwelling (pre)frail older adults. Participants with lower baseline physical capacity, who may be at greater risk losing independence, may benefit more from the programme, with potential improvements in physical functioning and capacity, frailty status, and participation limitations.

## Funding

The PromeTheus project is funded by the German Innovation Fund (‘New Forms of Care’) coordinated by the Innovation Committee of the Federal Joint Committee (in German: ‘Innovationsausschuss beim Gemeinsamen Bundesausschuss’, G‐BA; Grant No. 01NVF19020). The funder had no role in the study design, the collection, management, analysis and interpretation of data, writing the manuscript and the decision to submit this manuscript for publication.

## Conflicts of Interest

The authors declare no conflicts of interest.

## Supporting information


**Data S1:** Supporting Information.

**Table S1:** Effect of the PromeTheus programme after 6 months on primary and secondary outcomes.

**Table S2:** Adverse events experienced by participants throughout the trial.
