# Supplementary material for: Home‐Based Intervention to Prevent Functional Decline in (Pre)frail Older Adults: The PromeTheus Randomized Controlled Trial
Source: J Cachexia Sarcopenia Muscle. 2026 May 14;17(3):e70306. doi: 10.1002/jcsm.70306 (PMC13173293; doi:10.1002/jcsm.70306)
Supplement: Supplementary file 1 — Data S1: Supporting Information. [file JCSM-17-e70306-s001.docx]

**SUPPLEMENTAL REFERENCES**

1. Cesari M, Prince M, Thiyagarajan JA, De Carvalho IA, Bernabei R, Chan P, et al. Frailty: An Emerging Public Health Priority. *J Am Med Dir Assoc*. 2016;**17**(3):188-92.
2. Golliot F, Astagneau P, Cassou B, Okra N, Rothan-Tondeur M, Brücker G. Nosocomial infections in geriatric long-term-care and rehabilitation facilities: exploration in the development of a risk index for epidemiological surveillance. *Infect Control Hosp Epidemiol*. 2001;**22**(12):746-53.
3. Kannus P, Khan KM, Lord SR. Preventing falls among elderly people in the hospital environment. *Med J Aust*. 2006;**184**(8):372-3.
4. Kotsani M, Kravvariti E, Avgerinou C, Panagiotakis S, Bograkou Tzanetakou K, Antoniadou E, et al. The Relevance and Added Value of Geriatric Medicine (GM): Introducing GM to Non-Geriatricians. *J Clin Med*. 2021;**10**(14).
5. Treacy D, Hassett L, Schurr K, Fairhall NJ, Cameron ID, Sherrington C. Mobility training for increasing mobility and functioning in older people with frailty. *Cochrane Database Syst Rev*. 2022;**6**(6):Cd010494.
6. Morishita S, Tsubaki A, Nakamura M, Nashimoto S, Fu JB, Onishi H. Rating of perceived exertion on resistance training in elderly subjects. *Expert Rev Cardiovasc Ther*. 2019;**17**(2):135-142.
7. Izquierdo M, de Souto Barreto P, Arai H, Bischoff-Ferrari HA, Cadore EL, Cesari M, Chen LK, Coen PM, Courneya KS, Duque G, Ferrucci L, Fielding RA, García-Hermoso A, Gutiérrez-Robledo LM, Harridge SDR, Kirk B, Kritchevsky S, Landi F, Lazarus N, Liu-Ambrose T, Marzetti E, Merchant RA, Morley JE, Pitkälä KH, Ramírez-Vélez R, Rodriguez-Mañas L, Rolland Y, Ruiz JG, Sáez de Asteasu ML, Villareal DT, Waters DL, Won Won C, Vellas B, Fiatarone Singh MA. Global consensus on optimal exercise recommendations for enhancing healthy longevity in older adults (ICFSR). *J Nutr Health Aging*. 2025;**29**(1):100401.
8. Riebe D, Ehrman JK, Liguori G, Magal M. *ACSM's guidelines for exercise testing and prescription*. 10th ed. Wolters Kluwer Health; 2018.
9. Bull FC, Al-Ansari SS, Biddle S, Borodulin K, Buman MP, Cardon G, Carty C, Chaput JP, Chastin S, Chou R, Dempsey PC, DiPietro L, Ekelund U, Firth J, Friedenreich CM, Garcia L, Gichu M, Jago R, Katzmarzyk PT, Lambert E, Leitzmann M, Milton K, Ortega FB, Ranasinghe C, Stamatakis E, Tiedemann A, Troiano RP, van der Ploeg HP, Wari V, Willumsen JF. World Health Organization 2020 guidelines on physical activity and sedentary behaviour. *Br J Sports Med*. 2020;**54**(24):1451-1462.
10. Beauchamp MK, Schmidt CT, Pedersen MM, Bean JF, Jette AM. Psychometric properties of the Late-Life Function and Disability Instrument: a systematic review. *BMC Geriatr*. 2014;**14**:12.
11. Guralnik JM, Ferrucci L, Simonsick EM, Salive ME, Wallace RB. Lower-extremity function in persons over the age of 70 years as a predictor of subsequent disability. *N Engl J Med*. 1995;**332**(9):556-61.
12. Wijesuriya R, Moreno-Betancur M, Carlin JB, White IR, Quartagno M, Lee KJ. Multiple Imputation for Longitudinal Data: A Tutorial. *Stat Med*. 2025;**44**(3-4):e10274.
13. Lawson PJ, Flocke SA. Teachable moments for health behavior change: a concept analysis. *Patient Educ Couns*. 2009;**76**(1):25-30.
14. Jette AM, Haley SM, Coster WJ, Kooyoomjian JT, Levenson S, Heeren T, et al. Late life function and disability instrument: I. Development and evaluation of the disability component. *J Gerontol A Biol Sci Med Sci*. 2002;57(4):M209-16.
15. Campbell AJ, Robertson MC, Gardner MM, Norton RN, Tilyard MW, Buchner DM. Randomised controlled trial of a general practice programme of home based exercise to prevent falls in elderly women. *BMJ*. 1997;**315**(7115):1065-9.
16. Liu-Ambrose T, Davis JC, Best JR, Dian L, Madden K, Cook W, et al. Effect of a Home-Based Exercise Program on Subsequent Falls Among Community-Dwelling High-Risk Older Adults After a Fall: A Randomized Clinical Trial. *JAMA*. 2019;**321**(21):2092-100.
17. Ricke E, Dijkstra A, Bakker EW. Prognostic factors of adherence to home-based exercise therapy in patients with chronic diseases: A systematic review and meta-analysis. *Front Sports Act Living*. 2023; **5**:1035023.
18. Gómez-Redondo P, Valenzuela PL, Morales JS, Ara I, Mañas A. Supervised Versus Unsupervised Exercise for the Improvement of Physical Function and Well-Being Outcomes in Older Adults: A Systematic Review and Meta-analysis of Randomized Controlled Trials. *Sports Med*. 2024;**54**(7):1877-1906.
