# Supplementary material for: Home‐Based Intervention to Prevent Functional Decline in (Pre)frail Older Adults: The PromeTheus Randomized Controlled Trial
Source: J Cachexia Sarcopenia Muscle. 2026 May 14;17(3):e70306. doi: 10.1002/jcsm.70306 (PMC13173293; doi:10.1002/jcsm.70306)
Supplement: Supplementary file 2 — Table S1: Effect of the PromeTheus programme after 6 months on primary and secondary outcomes. [file JCSM-17-e70306-s002.docx]

**Table S1** Effect of the PromeTheus program after 6 months on primary and secondary outcomes

| **Outcome** | **Analysis** | **Model / Statistical test** | **Estimate (95% CI)** | ***P*-value** |
| --- | --- | --- | --- | --- |
| *Primary outcomes* | | | | |
| LLFDI function component | ITT | Multiple imputation two sample *t*-test^a^ | 1.38 (0.25, 2.51) | 0.017 |
|  | ITT | Linear mixed-effects model^a^ | 1.29 (0.19, 2.40) | 0.022 |
|  | ATP | Two sample *t*-test^a^ | 1.75 (0.60, 2.91) | 0.003 |
|  | ATP | Linear mixed-effects model^a^ | 1.77 (0.57, 2.96) | 0.004 |
| Life-Space Assessment | ITT | Multiple imputation two sample *t*-test^a^ | -0.01 (-3.95, 3.94) | 0.998 |
| (subordinated) | ITT | Linear mixed-effects model^a^ | -0.34 (-3.99, 3.31) | 0.855 |
|  | ATP | Two sample *t*-test^a^ | 0.07 (-3.93, 4.07) | 0.973 |
|  | ATP | Linear mixed-effects model^a^ | 0.06 (-3.96, 4.08) | 0.977 |
| *Secondary outcomes* | | | | |
| SF-LLFDI disability component |  |  |  |  |
| Frequency | ITT | Linear mixed-effects model^a^ | 0.28 (-0.39, 0.94) | 0.415 |
| Limitation | ITT | Linear mixed-effects model^a^ | 1.17 (-0.50, 2.84) | 0.171 |
| Frailty status | ITT | Multiple imputation ordinal logistic regression model^b^ | 1.33 (0.89, 1.98) | 0.165 |
| SPPB | ITT | Linear mixed-effects model^a^ | 0.58 (0.11, 1.05) | 0.015 |
| ^a^ Estimate is the difference in means (change between baseline and 6 months) between the intervention group and the control group.  ^b^ Estimate is the proportional odds ratio of improvement vs. no change or no change vs. deterioration after 6 months in the intervention group compared to the control group.  CI, confidence interval; LLFDI, Late-Life Function and Disability Instrument; ITT, intention-to-treat (*n* = 385); ATP, according-to-protocol (*n* = 275); SF-LLFDI, Short-Form Late-Life Function and Disability Instrument; SPPB, Short Physical Performance Battery. | | | | |
