# Supplementary material for: Home‐Based Intervention to Prevent Functional Decline in (Pre)frail Older Adults: The PromeTheus Randomized Controlled Trial
Source: J Cachexia Sarcopenia Muscle. 2026 May 14;17(3):e70306. doi: 10.1002/jcsm.70306 (PMC13173293; doi:10.1002/jcsm.70306)
Supplement: Supplementary file 3 — Table S2: Adverse events experienced by participants throughout the trial. [file JCSM-17-e70306-s003.docx]

**Table S2** Adverse events experienced by participants throughout the trial

| **Type**  **of event** | **IG (*n* = 196)** | |  | **CG (*n* = 189)** | | **Risk ratio**^a^  **(95% CI)** | **Rate ratio**^a^  **(95% CI)** |
| --- | --- | --- | --- | --- | --- | --- | --- |
|  | **No. of**  **participants (%)** | **No. of events**  **(rate per PY)** |  | **No. of**  **participants (%)** | **No. of events**  **(rate per PY)** |  |  |
| Any AE | 137 (69.9) | 421 (2.41) |  | 105 (55.6) | 262 (1.47) | 1.26 (1.08, 1.47) | 1.65 (1.27, 2.13) |
| SAE | 66 (33.7) | 100 (0.57) |  | 34 (18.0) | 46 (0.26) | 1.60 (1.19, 2.14) | 2.23 (1.44, 3.45) |
| IG, intervention group; CG, control group; PY, person-years, CI, confidence interval; AE, adverse event; SAE, serious adverse event.  Person-years were 174.5 for the intervention group and 178.7 for the control group.  ^a^ Estimates for the risk and rate ratios between the intervention group over the control group were obtained from Quasi-Poisson regression models. | | | | | | | |
